# The Interplay of Lung Surfactant Proteins and Lipids Assimilates the Macrophage Clearance of Nanoparticles

**DOI:** 10.1371/journal.pone.0040775

**Published:** 2012-07-10

**Authors:** Christian A. Ruge, Ulrich F. Schaefer, Jennifer Herrmann, Julian Kirch, Olga Cañadas, Mercedes Echaide, Jesús Pérez-Gil, Cristina Casals, Rolf Müller, Claus-Michael Lehr

**Affiliations:** 1 Department of Biopharmaceutics and Pharmaceutical Technology, Saarland University, Saarbrücken, Germany; 2 Department of Pharmaceutical Biotechnology, Saarland University, Saarbrücken, Germany; 3 Department of Biochemistry and Molecular Biology I, Universidad Complutense, Madrid, Spain; 4 CIBER de Enfermedades Respiratorias, Universidad Complutense, Madrid, Spain; 5 Helmholtz-Institute for Pharmaceutical Research Saarland (HIPS), Saarland University, Saarbrücken, Germany; University of Queensland, Australia

## Abstract

The peripheral lungs are a potential entrance portal for nanoparticles into the human body due to their large surface area. The fact that nanoparticles can be deposited in the alveolar region of the lungs is of interest for pulmonary drug delivery strategies and is of equal importance for toxicological considerations. Therefore, a detailed understanding of nanoparticle interaction with the structures of this largest and most sensitive part of the lungs is important for both nanomedicine and nanotoxicology. Astonishingly, there is still little known about the bio-nano interactions that occur after nanoparticle deposition in the alveoli. In this study, we compared the effects of surfactant-associated protein A (SP-A) and D (SP-D) on the clearance of magnetite nanoparticles (mNP) with either more hydrophilic (starch) or hydrophobic (phosphatidylcholine) surface modification by an alveolar macrophage (AM) cell line (MH-S) using flow cytometry and confocal microscopy. Both proteins enhanced the AM uptake of mNP compared with pristine nanoparticles; for the hydrophilic ST-mNP, this effect was strongest with SP-D, whereas for the hydrophobic PL-mNP it was most pronounced with SP-A. Using gel electrophoretic and dynamic light scattering methods, we were able to demonstrate that the observed cellular effects were related to protein adsorption and to protein-mediated interference with the colloidal stability. Next, we investigated the influence of various surfactant lipids on nanoparticle uptake by AM because lipids are the major surfactant component. Synthetic surfactant lipid and isolated native surfactant preparations significantly modulated the effects exerted by SP-A and SP-D, respectively, resulting in comparable levels of macrophage interaction for both hydrophilic and hydrophobic nanoparticles. Our findings suggest that because of the interplay of both surfactant lipids and proteins, the AM clearance of nanoparticles is essentially the same, regardless of different intrinsic surface properties.

## Introduction

Because of the potential use of nano-sized particulate systems for pharmaceutical or medical purposes, the inhalation and pulmonary deposition of nanoparticles is a vigorously discussed topic among nanomedicine researchers. However, the potential adverse health effects from nanoparticles are also of interest to nanotoxicologists, and the risk assessment of such systems in any application is a topic of increasing concern to both regulatory agencies and the public. The ongoing controversial discussions on the use of nanotechnology further emphasize the demand to carefully elucidate the biological fate, particularly the clearance, of inhaled nanoparticles in the human body [1]. In the lungs, clearance occurs either by the mucociliary escalator, by alveolar macrophages, or by translocation across the epithelial layer [2], [3].

From the perspective of both nanotoxicology and nanomedicine, it appears essential to understand the bio-nano interactions of inhaled nanomaterials [4], [5]. Non-cellular elements of the so-called air-blood barrier may play a key role here because this is the first biological matter that contacts nanoparticles after inhalation and deposition in the lungs. In the peripheral deep lungs, the so-called alveolar lining fluid, an ultra-thin layer that consists of an aqueous hypophase and a surface-active lipid-protein mixture, known as pulmonary surfactant, covers the epithelial cells. In the conducting airways, mucus – a rather thick layer with gel-sol characteristics – forms the main hurdle for deposited particles, when the underlying epithelium is the target [6], [7]. However, the pulmonary surfactant is continuous and spreads form the distal to the proximal lungs, where it consequently is positioned at the air-interface making it the most outer part of the air-blood barrier [8], [9].

Interactions between inhaled nanoparticles and the pulmonary surfactant system are of increasing interest and were the subject of several studies in the last years. However, recent work mainly focused on the influence nanoparticles exert on the biophysical function of pulmonary surfactant [10], [11], [12]. Such investigations are of great importance from a nano-safety perspective because they intend to find out if and how inhaled nanoparticles can alter the respiratory function. Astonishingly, however, there is still little known about the binding of surfactant biomolecules to nanoparticles and the potential for surfactant biomolecules to influence subsequent biological effects, such as the uptake by alveolar macrophages (AM); this uptake is the main clearance mechanism for particles from the peripheral lungs [4], [13], [14].

The composition of pulmonary surfactant is approximately 90% lipid and 10% protein [9]. Among the proteins, there are four surfactant proteins (SP-A, -B, -C, and -D), which have various biological functions. SP-B and SP-C are rather small peptides that interact with surfactant lipids and thereby contribute to proper biophysical surfactant functionality [15]. However, the pulmonary collectins SP-A (630 kD) and SP-D (520 kD) are of exceeding interest for bio-nano interactions because they fulfill important immunological functions by acting as opsonins and scavenger molecules [16]. Their occurrence at the air-liquid interface of alveoli ideally enables these two proteins to interact with and bind to airborne particulate matter deposited into the deep lungs and thus make first contact with pulmonary surfactant [17]. Furthermore, the fact that SP-A and SP-D can influence the uptake of particulate matter by AM also allocates them a key role in the clearance of inhaled nanoparticles [18].

We recently showed that nanoparticle uptake by AM can be triggered by SP-A, which indicated the importance of surfactant proteins as biomolecules determining the fate of inhaled nanoparticles in the deep lungs [19].

SP-D, however, is most likely of an equally great importance for protein-nanoparticle interactions because there is evidence that this protein can influence the phagocytosis of non-living particulate matter [20].

Hence, we studied and compared the potential of SP-A and SP-D to affect nanoparticle uptake by AM using MH-S cells, an immortalized murine AM cell line. Further, we analyzed the binding of SP-A and SP-D to various modified nanoparticles and their impact on colloidal stability. However, surfactant lipids, which constitute the major part of pulmonary surfactant, might modulate the action of surfactant proteins and were therefore also investigated.

## Results

To study the effect of SP-A SP-D on nanoparticle association with and uptake by macrophages, we chose magnetite nanoparticles (mNP) as a model system. All of the mNP were composed of the same magnetic core material but were decorated either with hydrophilic (starch, ST) or hydrophobic (phosphatidylcholine, PL) coatings. Thus, the mNP featured different intrinsic surface properties while maintaining comparable diameters and similar morphology (Table 1 and Fig. S1).

**Table 1:** Magnetic nanoparticle (mNP) characteristics and properties.

Name	Surface modification*	In MQ- water		In RPMI		In MQ- water	In RPMI
		Size (nm) ‡	PdI ‡	Size (nm) ‡	PdI ‡	Zeta-potential (mV) †	Zeta-potential (mV) †
ST-mNP	Starch	148.2±1.6	0.180±0.023	145.3±3.0	0.154±0.012	−4.15±0.22	−3.02±0.4
PL-mNP	Phosphatidylcholine	126.4±1.5	0.064±0.013	121.4±0.9	0.047±0.023	−34.5±0.23	−25.2±1.7

* Surface modification as indicated by the manufacturer (chemicell GmbH, Berlin, Germany).

‡ Hydrodynamic diameter and poly-dispersity index (PdI) as determined by dynamic light scattering using a Zetasizer Nano-ZS (Malvern Instruments). Data are shown as the mean ± SD (n = 3).

† Zeta-potential values were measured using the same machine and are displayed as the mean ± SD (n = 3).

**Figure 1:**
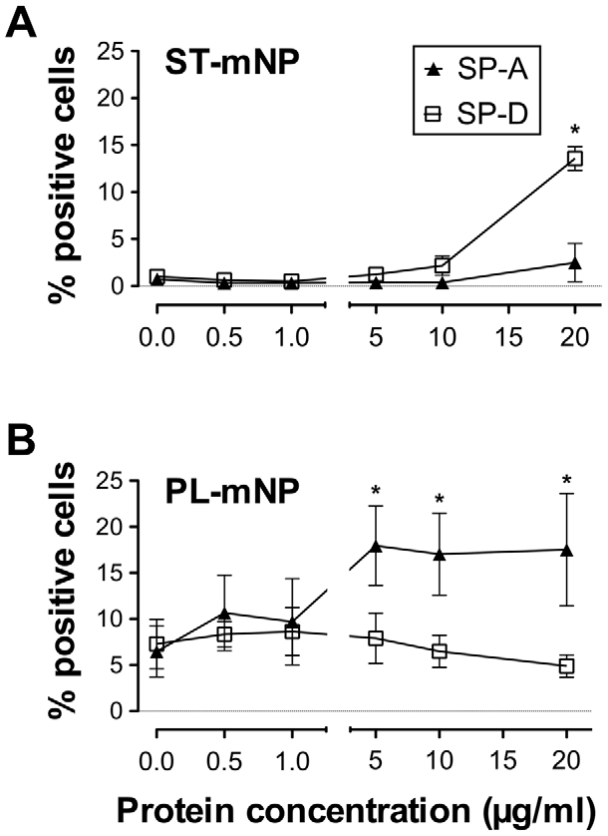
Association of nanoparticles with alveolar macrophages in presence of surfactant proteins as determined by flow-cytometry. (A) Starch-modified (ST) magnetite nanoparticles (mNP) or (B) Phosphatidylcholine-modified (PL) mNPs (1.532×10^10^/ml) were exposed to alveolar macrophages in the absence or presence of increasing surfactant protein concentrations and incubated for 90 min. Cells with Fl-1>10^1^ were considered as positive cells for nanoparticle association and uptake (expressed as percent of positive cells with respect to total cells) compared to control (cells only). Data shown as mean ± SD (n≥3). Asterisk indicates a significant difference with p<0.05, compared to mNP in the absence of proteins.

We found that SP-D mainly triggered the cellular association of ST-mNP (Fig. 1A), whereas SP-A predominantly increased the interaction of the PL-mNPs with cells (Fig. 1B). SP-A was able to significantly affect the cell association even at a concentration of 5 µg/ml, and this effect reached a plateau at the higher tested concentrations.

In contrast, SP-D- mediated effects were rather moderate, reaching statistical significance only at a four-fold higher protein concentration. However, no saturation in the cell binding of these nanoparticles was observed with the tested concentrations. The physiological concentration of SP-D in the alveolar lining fluid of non-smoking humans is approximately 1.3 µg/ml [21]; thus, concentrations above 20 µg/ml would be far beyond physiological relevance and were therefore not tested in this study.

**Figure 2:**
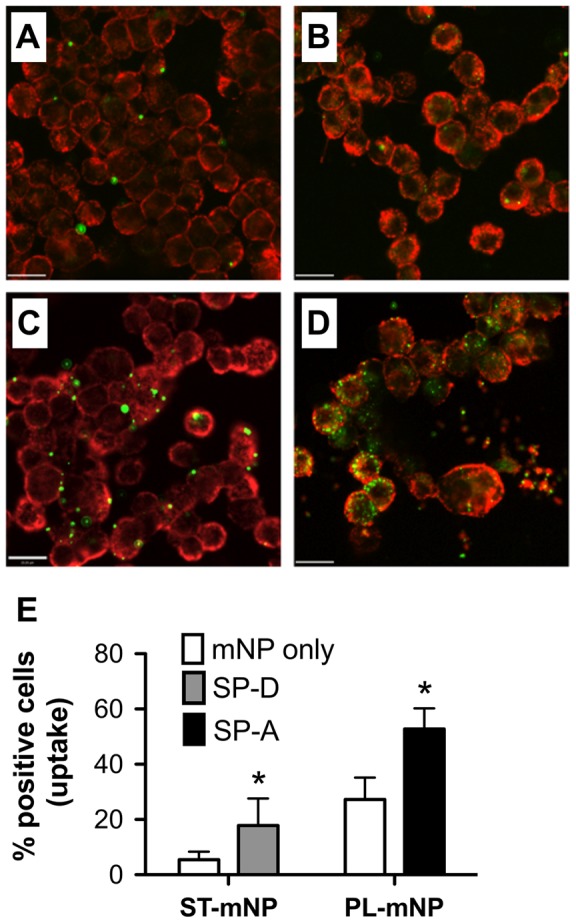
Nanoparticle uptake by alveolar macrophages in dependence on surfactant proteins as studied by confocal microscopy. Representative micrographs are shown for Starch-modified (ST) magnetite nanoparticles (mNP) (A, C) or Phosphatidylcholine-modified (PL) mNPs (B, D) (1.532×10^10^/ml, in green) after 90 min exposition to alveolar macrophages (MH-S cells; membrane in red) in absence (mNP only in buffer (A, B) or presence of (C) SP-D (10 µg/ml) or (D) SP-A (10 µg/ml). Scale bar indicates a distance of 20 µm. (E) Particle uptake determined by visual counting of cells with at least one nanoparticle internalized related to total cell count (% positive cells). Data shown as mean ± SD (n = 14 images). Asterisk indicates a significant difference with p<0.05.

Next, we used confocal microscopy to discriminate between cellular adherence and the internalization of nanoparticles (Fig. 2). Here, we focused on the effects of SP-D on ST-mNPs and SP-A on PL-mNPs, which corresponded to the previously observed increased cellular responses.

Micrographs recorded at the cellular equatorial plane reflected the trend from flow cytometry measurements: increased particle number per cell due to presence of surfactant proteins (Fig. 2A–D). Image analysis for the quantification of cellular uptake revealed that this trend was also accompanied by an increase in nanoparticle internalization (Fig. 2E), underscoring the role of surfactant proteins for uptake by AM. Both SP-A and SP-D most likely led to an alteration of the nanoparticles' outer appearance and thus, influenced the subsequent nanoparticle – cell interactions. Therefore, the binding of surfactant proteins and their impact on colloidal stability was investigated.

**Figure 3:**
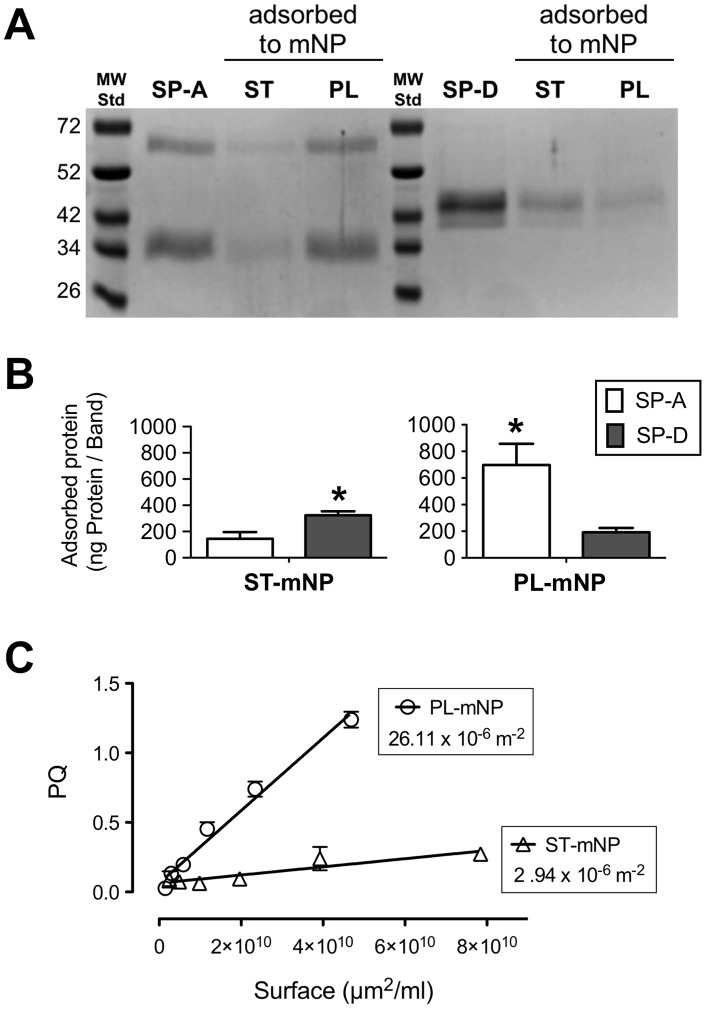
Adsorption of SP-A and SP-D to nanoparticles as affected by particle coating and hydrophobicity. (A) Representative SDS-PAGE gel displaying the adsorbed surfactant proteins (SP-A in the left lanes or SP-D in the right lanes) eluted from Starch- (ST) or Phosphatidylcholine-modified (PL) magnetite nanoparticles (mNP); MWStd stands for molecular weight standard. (B) Adsorbed amount of protein in nanogram of protein per band. (C) Plot of Rose Bengal – partitioning coefficient (PQ) as a function of total nanoparticle surface area (µm^2^). The slope of the lines was considered as a measure of relative nanoparticle hydrophobicity. Data shown as mean ± SD (n = 3). Significant differences between the two proteins are indicated with asterisks (p<0.05).

Adsorption experiments revealed a pronounced binding of SP-A to the PL-mNPs, whereas the ST-mNPs only showed a low binding to this protein. The same result was apparent when the samples were analyzed on Coomassie-stained SDS-PAGE gels (Fig. 3A), where the SP-A band corresponding to particle-bound protein was more intense for the PL-mNPs than for the ST-mNPs. Densitometry measurements of the protein signals (Fig. 3B) also confirmed this result. However, when the binding of SP-D was studied, we observed an opposite trend: SP-D a higher affinity for the ST-mNPs than for the PL-mNPs (Fig. 3B). Because hydrophobic interactions are described as the main contributor to protein-nanoparticle interactions [1], we performed a Rose Bengal (RB) assay to characterize the surface hydrophobicity of the nanoparticles [22]. The slopes deduced from plotting the partition coefficient of the hydrophobic dye RB as a function of total nanoparticle surface area are a relative measure for hydrophobicity (Fig. 3C). The PL-mNPs have a 10-fold steeper slope than the ST-mNPs; thus, higher amounts of RB were bound by the same total surface area of the PL-mNPs compared with the ST-mNP, indicating an overall more hydrophobic character for the PL-mNPs.

**Figure 4:**
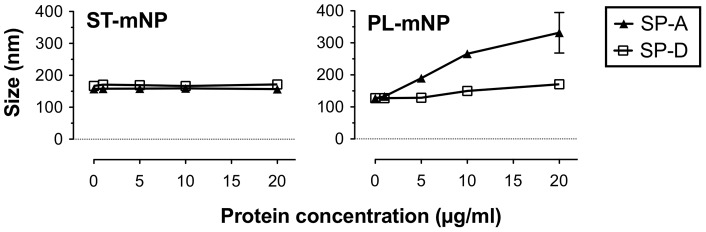
Influence of SP-A or SP-D on colloidal stability of nanoparticles. Increasing concentration of SP-A or SP-D were added to Starch-modified magnetite nanoparticles (ST-mNP; left) or Phosphatidylcholine-modified mNPs (PL-mNP; right). Hydrodynamic diameter (Z-average, size in nm) was determined subsequently after mixing using dynamic light scattering. Data shown as mean ± SD (n = 6).

In addition to protein adsorption, we used dynamic light scattering (DLS) to study the colloidal stability of the two nanoparticle types after incubation with SP-A or SP-D at concentrations corresponding to the ones used in the previous macrophage experiments (Fig. 4).

The hydrodynamic diameter of the ST-mNPs was not affected by SP-A (167.1±4.5 nm) and only slightly increased compared with the absence of protein when measured after incubation with SP-D (171.6±6.7 nm at 20 µg/ml). In the case of PL-mNPs, however, measurements revealed the formation of agglomerates in the presence of SP-A at a concentration of 10 µg/ml or higher, whereas SP-D did not cause such an effect. The same trend was also observed when the samples were analyzed again at later time-points, showing even higher sizes for the PL-mNPs (data not shown).

Overall, we discovered that the interplay of AM with mNPs can be influenced by either SP-A or SP-D because of their particle adsorption and their impact on particle stability. These findings indicate their relevance for bio-nano interactions in the alveolar lining fluid.

**Figure 5:**
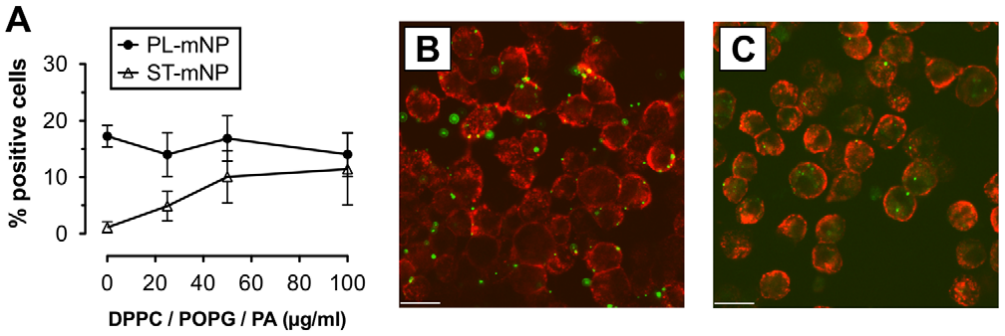
Effect of surfactant lipids on the association of nanoparticles with alveolar macrophages. (A) Starch- (ST) or Phosphatidylcholine-modified (PL) magnetite nanoparticles (mNP; 1.532×10^10^/ml) were incubated with alveolar macrophages (AM; MH-S cells) for 90 min in the absence or presence of 25, 50 or 100 µg/ml (total lipids) of surfactant-like membrane vesicles composed of dipalmitoyl-glycero-phosphocholine (DPPC), palmitoyl-oleoyl-glycero-phosphoglycerol (POPG) and palmitic acid (PA). Cells with Fl-1>10^1^ were considered as positive cells for nanoparticle association and uptake (plotted as percent of positive cells with respect to total cells) compared to control (cells only). Data shown as mean ± SD (n = 6). Images are representative micrographs of AM (membrane in red) with ST-mNPs (B) or PL-mNPs (C) (in green), both in the presence of 100 µg/ml DPPC/POPG/PA. Scale bar indicates a distance of 20 µm.

Pulmonary surfactant, however, is a complex mixture composed of both lipids and proteins [9]. Hence, inhaled nanoparticles – in contrast to intravenously applied particles – will never come into contact with proteins only. In fact, as soon as nanoparticles contact the pulmonary surfactant system, they will also interact with surfactant lipids. To address this issue, we prepared small unilamellar vesicles composed of dipalmitoyl-glycerophosphocholine, palmitoyl-oleoyl-glycerophosphoglycerol and palmitic acid (DPPC/POPG/PA), resulting in membrane structures that are known to have pulmonary surfactant-like properties [23]. Then, we studied nanoparticle uptake by AM in the presence of increasing DPPC/POPG/PA vesicle concentrations to explore the effects of surfactant lipids in a well-defined system (Fig. 5).

The PL-mNPs had much more cell interaction than the ST-mNPs when studied in buffer only (0 µg/ml DPPC/POPG/PA) and also showed the same extent of interaction with all of the DPPC/POPG/PA concentrations that were tested (Fig. 5A). However, the cell binding of the ST-mNPs grew more pronounced with increasing DPPC/POPG/PA concentrations, reaching a similar level of cell interaction as the PL-mNPs at 100 µg/ml. This trend was also confirmed by confocal microscopy (Fig. 5B and C).

**Figure 6:**
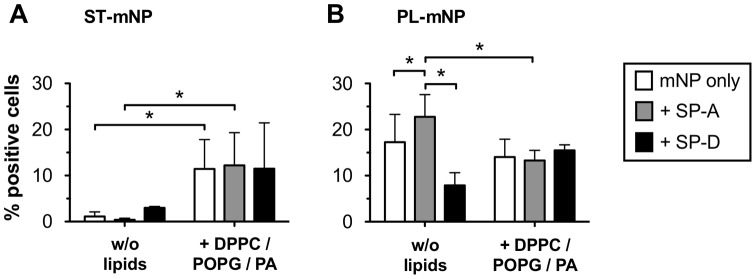
Modulation of SP-A – and SP-D – mediated macrophage association of mNPs by surfactant lipids. (A) Starch- (ST) or Phosphatidylcholine-modified (PL) magnetite nanoparticles (mNP; 1.532×10^10^/ml) were incubated with alveolar macrophages (AM; MH-S cells) for 90 min in the absence (mNP only in buffer) or presence of DPPC/POPG/PA vesicles (100 µg/ml) with and without 5 µg/ml SP-A or SP-D, respectively. Cells with Fl-1>10^1^ were considered as positive cells for nanoparticle association and uptake (% positive cells). Data shown as mean ± SD (n = 6). Asterisk indicates a significant difference with p<0.05.

Nonetheless, in the *in vivo* situation, surfactant lipids are highly associated with surfactant proteins. Consequently, we also tested the DPPC/POPG/PA vesicles supplemented with either SP-A or SP-D, which resulted in physiological lipid-protein ratios (Fig. 6).

Surprisingly, neither SP-A nor SP-D influenced the association of the mNPs with cells when studied in combination with DPPC/POPG/PA; the levels of macrophage binding were actually similar to those in the absence of the proteins (Fig. 6A, “mNP only”). For the ST-mNPs, the amount of cellular interaction in the presence of DPPCPOPG/PA increased compared with the conditions without these lipids (Fig. 6A), as observed in the experiments above. In the case of the PL-mNPs, however, the enhancement of SP-A decreased, resulting in similar levels for all three conditions in the presence of DPPC/POPG/PA (Fig. 6B). In fact, the presence of surfactant lipids seemed to neutralize the effect observed with SP-A only (w/o); this effect was similar to the cell binding of the ST-mNPs in the presence of both surfactant proteins and surfactant lipids.

**Figure 7:**
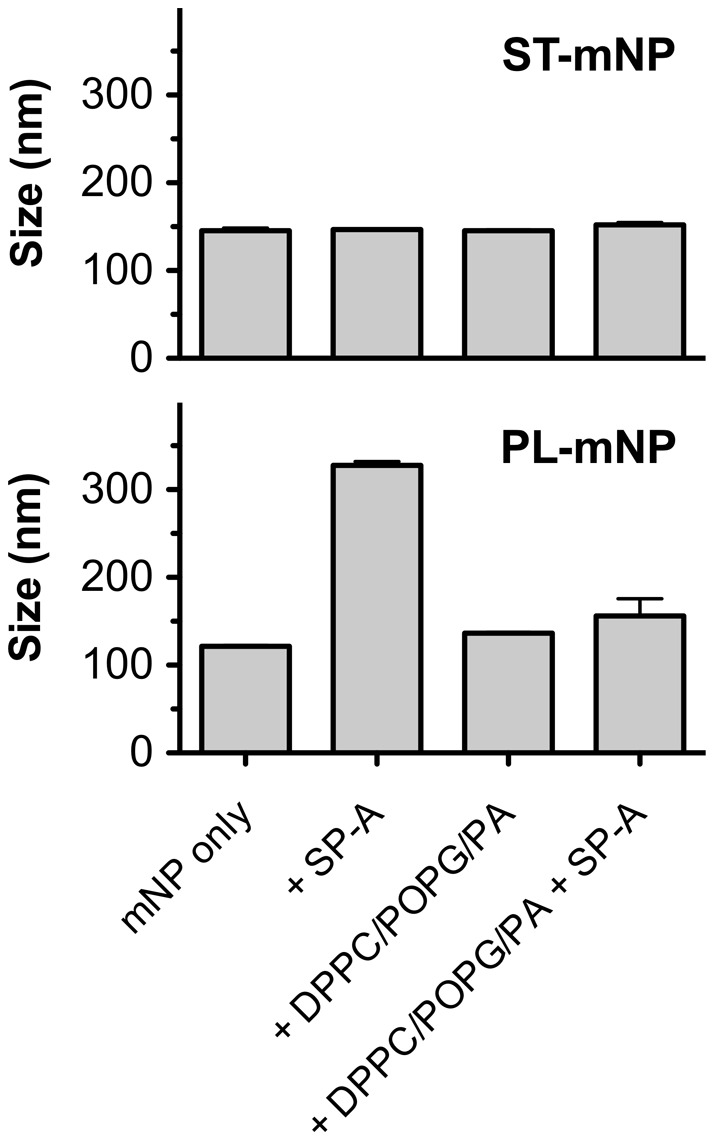
Influence of surfactant lipids on colloidal stability of nanoparticles in presence and absence of SP-A. Starch-modified (ST) magnetite nanoparticles (mNPs; top) or Phosphatidylcholine-modified (PL) mNPs (bottom) were studied in absence (mNP only), or in presence of DPPC/POPG/PA (100 µg/ml total lipids), with or without SP-A (5 µg/ml). Hydrodynamic diameter (Z-average, in nm) was determined subsequently after mixing using dynamic light scattering. Data shown as mean ± SD (n = 3).

However, surfactant lipids (DPPC/POPG/PA) did not cause any formation of particle agglomerates, which was confirmed by size measurements of the nanoparticles in the presence of DPPC/POPG/PA (Fig. 7). For SP-A and the PL-mNPs, particle agglomeration was even prevented, suggesting that surfactant lipids obviously can account for the maintenance of colloidal stability.

For the *in vivo* situation, we must admit that these findings were obtained upon exposure of the mNPs to a mixture of lipids and surfactant proteins, which is still an artificial model for pulmonary surfactant. Hence, we also studied macrophage uptake of nanoparticles in the presence of native surfactant (NS), containing physiological ratios of surfactant lipids, hydrophobic surfactant proteins and SP-A and therefore being of higher physiological relevance [24]. The presence of SP-A as the protein of interest in the NS preparations was confirmed by MALDI-ToF mass spectrometric analysis after trypsin digestion of the respective gel band. Because of the complexity of the NS, this isolate is often considered a biological standard to evaluate exogenous surfactant preparations [25], [26]. However, because NS lacks SP-D, we additionally tested NS formulations spiked with amounts of SP-D that were in a physiological ratio to SP-A (∼10∶1 (w/w) SP-A to SP-D).

**Figure 8:**
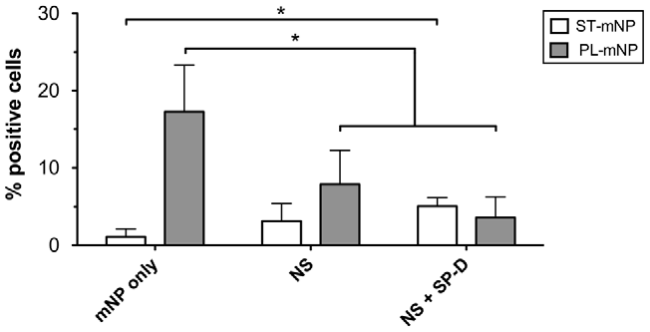
Association of nanoparticles with alveolar macrophages in dependence on native surfactant. Starch- (ST) or Phosphatidylcholine-modified (PL) magnetite nanoparticles (mNP; 1.532×10^10^/ml) were incubated with alveolar macrophages (MH-S cells) for 90 min in absence (mNP only in buffer) or presence SP-A containing Native Surfactant (NS; 100 µg/ml total protein) with and without 6 µg/ml SP-D (NS + SP-D). Per cent of cells with Fl-1>10^1^ were considered as positive cells for nanoparticle association and uptake (% positive cells). Data shown as mean ± SD (n≥8). Asterisk indicates a significant difference with p<0.05.

For the ST-mNPs, the NS lead to a considerable increase in nanoparticle uptake (Fig. 8). Although the percentage of positive cells was lower overall in comparison to the preparations including DPPC/POG/PA (Fig. 6A), the previously observed enhancing effect of surfactant lipids was thereby confirmed. NS spiked with SP-D (NS+SP-D) further increased the association; the percentage of positive cells was significantly higher compared with that for mNPs in buffer only (mNP only).

In the case of the PL-mNPs, however, cell binding was gradually equalized to that of the ST-mNPs and rather low when investigated with either NS alone or NS supplemented with SP-D. Furthermore, the uptake of the PL-mNPs was significantly decreased in the presence of NS when compared to mNP only, regardless if spiked with SP-D or not. Astonishingly, both types of mNPs demonstrated a similar extent of macrophage interaction when studied in NS spiked with SP-D, which was the most complex pulmonary surfactant model used in this study.

## Discussion

In the present study, we demonstrated that the pulmonary collectins SP-A and SP-D both can significantly trigger the interaction of nanoparticles with AM (Fig. 1 and 2). Additionally, we showed that the observed cellular effects were preceded by protein adsorption, leading to an alteration of the nanoparticle surface properties, and sometimes also causing particle agglomeration. With respect to the latter, the present study essentially confirmed the data we previously reported for SP-A [14], [19]. Such protein-mediated effects are thus the consequence of their adsorption to the nanoparticles, resulting in the formation of a protein corona around the nanoparticles, which further determines their biological fate [27], [28]. Here, we observed a pronounced binding of SP-A to the PL-mNPs, whereas SP-D preferentially adsorbed to the ST-mNPs. These results indicate that surfactant protein binding is crucially dependent on the nanoparticle surface material because both mNP types interacted differently with the respective proteins.

From a broader perspective, these findings might indicate the distinct differences between these two surfactant proteins in their binding to biological structures [17]. Indeed, such variations in pattern recognition might also exist for the interaction with deposited nanoparticles. The tendency of the pulmonary collectins SP-A and SP-D to bind nanoparticles might be determined by both the sum of general physicochemical interactions, such as electrostatic or hydrophobic interactions, and also the material-specific molecular interactions. From electrostatic surface potential modeling it is known that SP-A is more hydrophobic than SP-D [29]. Thus, whereas SP-A might preferably bind lipophilic patterns, SP-D might interact with more polar substrates [17]. This hypothesis is consistent with our data from the RB assay: SP-A preferentially bound lipophilic PL-mNPs and SP-D tended to adsorb to the rather hydrophilic ST-mNPs (Fig. 3B and C). Furthermore, SP-A is able to bind lipids such as lipopolysaccharide [17] and DPPC [30], the major phospholipid in pulmonary surfactant [9]. Additionally, SP-D was shown to bind pollen starch granules [20]. However, additional study will be necessary to further elucidate such interactions on a molecular level.

Nonetheless, our data corroborate that the adsorption of SP-A or SP-D to nanoparticles can occur and is largely governed by the material and therefore surface properties of the nanomaterials. Moreover, we provide evidence that SP-A or SP-D can each have a striking impact on the macrophage clearance of nanoparticles.

These triggering effects could include protein-mediated agglomeration of the nanoparticles. SP-A, for instance, promotes the aggregation of phospholipid vesicles and is self-aggregates under certain conditions [30], [31]. Hence, the agglomeration of the PL-mNPs observed in this study might be partly explained by taking these attributes into account (Fig. 4). Further, SP-A-induced agglomeration could lead to both higher cell deposition and enhanced phagocytosis, the latter of which is a size-dependent process [13].

Moreover, one could also think of a receptor-mediated mechanism exerted by the adsorbed surfactant proteins that would facilitate the endocytotic ingestion of a nanoparticle; the ingestion could be caused by the interaction with SP-A- or SP-D-binding receptors on the cellular surface of AM, such as SPR210 or glycoprotein-340, respectively [16], [32]. In summary, the increased uptake by AM most likely originates from both mechanisms occurring simultaneously and cooperatively; SP-A and SP-D most likely act as “second messengers” for nanoparticles, finally fulfilling their physiological function, which is binding to foreign material and promoting its clearance from the lungs.

However, when particle clearance was studied in the presence of surfactant lipids, the effects exerted by the surfactant proteins were significantly modulated, eventually causing even opposite effects. On the one hand, we found that the uptake of the ST-mNPs by AM was increased in the presence of surfactant lipids, regardless if studied with synthetic or naturally derived surfactant preparations (Fig. 5A and 6A). These data suggest that nanoparticles, when surrounded and coated with surfactant lipids, can presumably be internalized by AM to an elevated degree, providing a more rapid clearance. In this respect, the PL-mNP data support such a hypothesis because these particles already bear a phosphatidylcholine-coating and showed an overall enhanced interaction with AM compared to the ST-mNPs (Fig. 5A, 0 µg/ml DPPC/POPG/PA). A similar influence of surfactant lipids was also reported in an earlier study by Stringer et al., who studied how titanium dioxide microparticle uptake by AM depended on Survanta®, a complex surfactant lipid extract [33].

On the other hand, surfactant lipids seemed to counterbalance the effect of SP-A on the interaction of PL-mNPs with macrophages (Fig. 6B and 8). These findings indicate that certain surfactant components have an inhibiting effect on AM activities. In fact, it is known that surfactant lipids can suppress the stimulation of immune cells to maintain homeostasis in the host defense system at the air-interface [34]; these effects also seem to play an important role in macrophage clearance of larger particulate matter [35]. Therefore, a decrease in cellular interaction as observed with PL-mNPs could be explained as an inhibiting effect of free surfactant lipids. This behavior would also account for the prior finding where no further increase in cellular interaction was observed for the PL-mNPs in the presence of any of the DPPC/POPG/PA concentrations studied (Fig. 5A). In this context, it is also likely that free surfactant lipids act as competitive binding partners for SP-A, preventing the protein from sufficient nanoparticle binding and cross linking and therefore enhanced particle agglomeration (Fig. 7). Additionally, the reduced interaction of the PL-mNPs in the presence of NS (Fig. 8) could also be linked to the macromolecular structure of the large NS-vesicles, which might act as an extracellular membranous barrier that partially hinders nanoparticles from contact with cells.

Both the enhancing and inhibiting effects of surfactant lipids on nanoparticle uptake can occur and most likely interfere with each other. Anyhow, both types of nanoparticles interacted to an approximately equivalent extent with AM in the presence of NS, regardless of their different surface modifications. Additionally, surfactant lipid membranes composed of DPPC/POPG/PA and NS- preparations were also shown to modulate the effects exerted by SP-A or SP-D only (Fig. 6 and 8). These observations might also be related to the counterbalancing interplay of proteins and lipids in pulmonary surfactant [34]. Different situations might occur in different locations of the airways. The airspaces in the upper airways could be particularly enriched in collectins and other innate defense molecules, promoting the capture of spurious particles by macrophages; whereas in the alveolar spaces, which are rich in surfactant, the immuno-modulatory properties of phospholipids could dominate, preventing inflammation but increasing the persistence, both in amplitude and duration, of nanoparticles in the extracellular material.

Nevertheless, we could show that proteins can exert significant effects on the biological fate of nanoparticles (Fig. 1). Thus, our results support the protein corona theory. According to this theory, a dynamic layer of protein is formed around nanoparticles that defines their biological identity once in contact with living systems [27]. Until now, this theory has primarily considered the interactions of nanoparticles with components of blood plasma.

The major constituent of pulmonary surfactant, however, is lipid. In fact, our data demonstrate that surfactant lipids can dramatically modulate protein-mediated effects. Accordingly, surfactant lipids most likely influence the subsequent cellular effects within the alveolar lining fluid, making them an important factor for pulmonary bio-nano interactions. One might speculate that the surface association of surfactant proteins and lipids could result in a rather complex mixed corona around nanoparticles upon first contact with pulmonary surfactant. A detailed characterization of such a “pulmonary corona” is still an indispensable topic and will be the subject of future investigations.

Apart from the question of whether a specific corona is actually formed or not, the lung surfactant components have a significant influence on AM clearance of nanoparticles: Although the AM uptake of pristine nanoparticles with different surface properties (e.g., hydrophilic vs. hydrophobic) differed, it became essentially the same in the presence of NS (i.e., under the conditions most closely resembling the *in vivo* situation). One might speculate that the surface association of surfactant proteins and lipids could result in a rather complex mixed corona around nanoparticles upon first contact with pulmonary surfactant. Because surfactant lipids are obviously highly relevant to bio-nano interactions in the lungs, we therefore suggest an expansion of the protein corona theory to include lipids as another essential factor influencing the pulmonary corona. Its detailed characterization is an indispensable issue to address in future investigations. We believe that these findings are of general importance with respect to pulmonary drug delivery and inhalation toxicology. On the one hand, such assimilation might question the hitherto pursued targeting strategies using nanoparticles because surface-ligands meant for cellular recognition could be masked by pulmonary surfactant components and therefore inactivated. On the other hand, the interplay of the surfactant proteins and lipids as a trigger for AM clearance creates a highly effective biological barrier, protecting the human body against potential noxious effects from any kind of inhaled nanomaterials or pathogens.

## Materials and Methods

### Materials

Magnetite nanoparticles (mNPs; nano-screenMAG, magnetite core with starch- (ST-mNP) or phosphatidylcholine- (PL-mNP) matrix) were purchased from chemicell GmbH (Berlin, Germany). The nanoparticles were ordered with a yellow-green fluorescence color (Ex: 476 nm, Em: 490 and 515 nm) and a hydrodynamic diameter of 150 nm as indicated by the manufacturer. Human surfactant protein A (SP-A) from alveolar proteinosis patients was isolated and characterized as described by Sanchez-Barbero et al. [36]. The protein concentration was determined using a bicinchoninic acid assay kit according to the manufacturer's instructions (Sigma, Munich, Germany). Recombinant human surfactant protein D (SP-D) was obtained from R&D systems (Minneapolis, MN, USA). The lyophilized protein was reconstituted at 100 µg/ml in PBS (Dulbecco's Phosphate Buffered Saline; Sigma) prior to use or stored as aliquots at −20°C. Synthetic 1,2-dipalmitoyl-sn-glycero-3-phosphocholine (DPPC; PC 16∶0/16∶0) and 1-palmitoyl-2-oleoyl-sn-glycero-3-phospho-rac-glycerol (POPG; PG 16∶0/18∶1) were received from Lipoid GmbH (Ludwigshafen, Germany). Palmitic acid (PA) was purchased from Sigma. Native pulmonary surfactant was isolated from porcine lungs obtained from a local slaughterhouse as described elsewhere [37]. The cell culture reagents were obtained from Gibco (Invitrogen, Carlsbad, CA, USA) if no other source is stated. All other chemicals and reagents were purchased from Sigma unless indicated otherwise.

### Isolation of surfactant protein A and native surfactant

Native pulmonary surfactant from adult pig lungs was obtained as described elsewhere [37], [38]. Briefly, cell-free bronchoalveolar lavage (BAL) was centrifuged at 100,000 g for 2 h at 4°C. The obtained large surfactant aggregates in the pellet were subsequently purified from the blood components using a NaBr density-gradient centrifugation at 116,000 g for 2 h at 4°C. The total phospholipid was determined by phosphorus analysis as described by Rouser et al. [39]. The total protein concentration of native surfactant was determined using a BCA assay kit (Sigma) according to the manufacturer's instructions. Human SP-A was isolated from BAL of patients with alveolar proteinosis using a sequential butanol and octylglucoside extraction [31], [36]. The purity of SP-A was checked by one-dimensional SDS-Page in 12% acrylamide under reducing conditions. The oligomerization state of SP-A was assessed by electrophoresis under non-denaturing conditions, electron microscopy, and analytical ultracentrifugation as reported elsewhere [36], [40]. The biological activity of isolated SP-A was assayed by testing its ability to self-associate and to induce aggregation of phospholipids and bacterial lipopolysaccharides in the presence of calcium at 37°C as described elsewhere [36], [40].

### MALDI-ToF mass spectrometry

For mass spectrometry analysis of SP-A in native surfactant, the samples were separated by SDS-PAGE under same conditions as the Western Blotting. After electrophoresis, the proteins bands were visualized using PageBlue colloidal Coomassie staining solution (Fermentas). The protein of interest (34 kD band) was excised from the gel automatically using SpotPicker (GE Healthcare). The slices were then washed with distilled water and destained with a 1∶1 mixture of 40 mM ammonium bicarbonate and acetonitrile (ACN). After a 15 min incubation with 100% ACN gel, the plugs were completely dried and finally rehydrated in a minimal volume of 40 mM ammonium bicarbonate containing 25 ng/ml trypsin and incubated overnight at 37°C. The in-gel digests were concentrated and desalted using ZipTipC18 (Millipore) by elution with 50% (v/v) ACN and 0.1% (v/v) trifluoric acid (TFA). Aliquots of the peptide solution (0.7 μl) prepared from the protein spots were mixed with 0.4 μl of α-Cyano-4-hydroxy cinnammic acid (CCA, 5 mg/ml in 50% (v/v) ACN and 0.1% (v/v) TFA) on a stainless steel target using the dried droplet method [41]. Selected peptides of PMF obtained by MALDI-MS in reflector mode were further fragmented by MALDI-PSD using a 4800 MALDI TOF/TOF™ Analyzer (Applied Biosystems, Carlsbad, CA, USA). Peptide mass standards (Applied Biosystems) were used for internal calibration of the mass spectra. NCBI database searching resulted in a protein score of 62 for human pulmonary surfactant-associated protein A (GI: 257467612). The coverage of MS-MS-identified human SP-A peptides homologous to porcine SP-A was approximately 27%.

### Cell culture

Murine AM (MH-S; ATCC CRL-2019, Wesel, Germany) were cultured under adherent conditions in cell culture medium (RPMI 1640 with L-glutamine supplemented with 10% (v/v) fetal calf serum (FCS; PAN Biotech, Aidenbach, Germany), 1% (v/v) HEPES, 25 mM D-glucose, 18 mM sodium bicarbonate (Merck), 1 mM sodium pyruvate and 0.05 mM β-mercaptoethanol). For the uptake experiments, 2×10^5^ cells per ml were seeded in 24-well plates (for flow-cytometry studies; Greiner Bio-One, Frickenhausen, Germany) or in 24-well imaging plates (for confocal microscopy studies; zell-kontakt®, Nörten-Hardenberg, Germany) and cultivated for 48 h in cell culture medium. The cells were incubated at 37°C under a humidified 5% CO_2_ atmosphere.

### Flow cytometry-based cell association assay

Test formulations were prepared as follows: 10 µl of ST- or PL-mNP from a 550 µg/ml stock suspension in RPMI was added to 190 µl of RPMI supplemented with surfactant proteins (SP-A or SP-D at 0.5, 1, 5, 10 or 20 µg/ml), surfactant lipids (0, 1, 10, 25, 50 or 100 µg/ml total lipids) or native pulmonary surfactant (100 µg/ml total protein). Prior to each uptake experiment, the cells were washed with RPMI (w/o FCS) to remove serum proteins and equilibrated for 30 min at 37°C. After this pre-incubation, RPMI was replaced by the nanoparticle test formulation (1.532×10^10^ mNPs per ml) and incubated under gentle shaking (150 rpm; IKA MTS 2/4 digital, IKA, Staufen, Germany) at 37°C for 90 min. All of the experiments were performed in the absence of FCS to identify effects mediated by the particular surfactant proteins. After nanoparticle expositon, the cells were washed twice with PBS and harvested for flow cytometric analysis using a FACSCalibur (Becton Dickinson, Franklin Lakes, NJ, USA). Five to ten thousand events were acquired in a gate based on the forward and side scatter. The level of mNP-macrophage interaction was measured as an increase of the fluorescence in the Fl-1 channel and are subsequently referred to as percent positive cells. Percent values of events with Fl-1 above 10^1^ of cells only (w/o nanoparticles) were subtracted from each sample as background. The data analysis was conducted with FlowJow (Version 8.8.6; Tree Star Inc., Ashland, OR, USA). The experiments were performed in duplicates and repeated at least three times.

### Confocal microscopy-based uptake study

The visualization experiments were conducted in analogy to the flow cytometry-based assays (see above). After 90 min of incubation at 37°C under gentle shaking, the cells were washed twice with PBS. The cell membranes were subsequently stained with Rhodamine riccinus communis agglutinin I (RRCA; 1∶400 in PBS; Vector Laboratories, Burlingame, CA, USA) at 37°C for 10 min, and the cells were fixed with formaldehyde (4% (v/v) in PBS) for 10 min after two intermediate PBS washing steps. A Zeiss LSM 510 with META detector (Carl Zeiss AG, Oberkochen, Germany) equipped with a 40x water immersion objective was used to visualize the specimens. Micrographs with more than 10 cells recorded in the equatorial plane of the cells were used for a quantitative discrimination between internalized and adherent nanoparticles. The cells were visually counted for each micrograph (n = 7 micrographs per condition), and cells with at least one nanoparticle located inside the membrane boundary were expressed as percent uptake-positive cells of total cell number. The images were analyzed using Volocity LE (Version 5.3.1; Perkin Elmer, Waltham, MA, USA). The experiments were performed in duplicates and repeated two times.

### Protein adsorption study

To investigate the adsorption of SP-A and SP-D to nanoparticles, 20 µl of ST-mNP or PL-mNP as suspensions of 2000 µg/ml in RPMI (RPMI 1640 with L-glutamine, supplemented with 1% (v/v) HEPES, 25 mM D-glucose, 18 mM sodium bicarbonate (Merck), and 1 mM sodium pyruvate) was incubated with 180 µl of protein solution (10 µg/ml in RPMI) for 10 min at 37°C. After incubation, the nanoparticle-protein complexes were removed from the supernatants using magnetic separation (Magna GrIP™ Rack, Millipore). The nanoparticle pellets were washed by resuspending with 200 µl of RPMI, followed by magnetic separation. After removal of the supernatants, pellets were resuspended in 40 µl of SDS-PAGE sample buffer (25% (w/v) glycerol, 60 mM Tris-HCl pH 6.8, 2% (w/v) SDS (Serva, Heidelberg, Germany), 0.1% (w/v) Bromophenol Blue (Merck, Darmstadt, Germany), and 5% (v/v) β-mercaptoethanol in MQ-water (Millipore Corporation, Billerica, MA, USA)) and heated in a water bath at 95°C for 5 min to elute adsorbed proteins from the particles. The protein samples were separated on 10% SDS-polyacrylamide gels at 130 V for 1 h using a MiniProtean Tetracell (BioRad, Munich, Germany). The gels were subsequently stained with colloidal Coomassie (PageBlue, Fermentas, St. Leon-Rot, Germany). Gel analysis and protein quantification using SP-A or SP-D standards (1000 ng band) was conducted with Image Lab (Version 4.0 build 16; BioRad, Munich, Germany). The experiments were repeated three times.

### Nanoparticle Size and Zeta-potential

All of the tested nanoparticles were characterized in terms of the hydrodynamic diameter and size distribution using DLS. Briefly, 10 µl of mNP (550 µg/ml in RPMI) were mixed with 200 µl of MQ-water or test medium (RPMI), supplemented with 1, 5, 10 or 20 µg/ml SP-A or SP-D, or 100 µg/ml surfactant lipids. Then, the samples were diluted by the addition of 800 µl of RPMI and subsequently measured using a Zetasizer Nano-ZS (Malvern Instruments, Malvern, UK). The zeta-potentials were determined using the same apparatus. The experiments were repeated at least twice.

### Rose Bengal Assay

The surface hydrophobicity of the tested nanoparticles was assessed using the Rose Bengal (RB, Sigma) assay as described by Müller et al. [22]. Briefly, 150 µl of mNP suspension (5 mg/ml to 78.125 µg/ml (1∶1 dilutions) in PBS, pH 7.4) was incubated with 150 µl of RB (40 µg/ml in PBS) for 30 min at RT. As a control, 150 µl of RB (40 µg/ml) were incubated with 150 µl of PBS only. After incubation, the samples were centrifuged at 10,835 g at 4°C for 20 minutes. The supernatants were carefully removed and the absorption at 542 nm was measured using an Infinite 200 M multimode microplate reader (Tecan Group Ltd., Männedorf, Switzerland). The RB concentration in supernatants (free amount in dispersion medium) was determined using a RB calibration curve (40-0.625 µg/ml). The nanoparticle-adsorbed amount of RB was calculated according to equation 1:
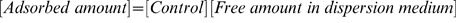
(1)


Subsequently, the partition coefficient PQ of RB between the nanoparticle surface and the dispersion medium was calculated for each nanoparticle concentration using equation 2:
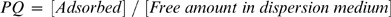
(2)


The PQ was plotted against the total nanoparticle surface (µm^2^), assuming spherical particles with diameters as determined by DLS. The slope of the resulting line was used as a measure for the relative hydrophobicity.

### Preparation of Surfactant Lipid Vesicles

Surfactant lipids (DPPC, POPG, PA) were dissolved in chloroform/methanol (3∶1, v/v) to prepare stock solutions with a concentration of 20 mg/ml (w/v). The appropriate amounts of surfactant lipid stock solutions were mixed to obtain a final weight ratio of 28∶9∶5.6 (DPPC/POPG/PA) with a total lipid mass of 1 mg. The organic solvents were evaporated under nitrogen with subsequent centrifugation under reduced pressure for 1 h using a Concentrator plus (Eppendorf, Hamburg, Germany). Subsequently, the dried lipid mixture was rehydrated with 1 ml of RPMI on a Thermomixer (Eppendorf, Hamburg, Germany) at 800 rpm and 48°C for 1 h. The resulting multilamellar suspensions were sonicated for 3 min (bursts of 0.6 s with 0.4 s between bursts) using a 2 mm microtip with a Branson Digital Sonifier 250 (Branson Ultrasonics, Danbury, CT, USA) at 10% amplitude to produce unilamellar vesicles. The surfactant lipid vesicles were characterized in terms of size distribution; the mean peak size was 50.7 nm as determined by dynamic light scattering.

### Statistics

The differences in protein adsorption were analyzed using Student's t-test. The differences in nanoparticle-macrophage interaction studies were analyzed using one-way ANOVA followed by the Newman-Keuls posthoc tests using Graphpad 5 for Mac. For all of the tests, p<0.05 was considered as a significant difference.

## Supporting Information

Figure S1 **Microscopic images of used magnetic nanoparticles** (**mNPs**)**.** SPM- (left) and SEM-images (right) of starch- (ST) and phosphatidylcholine- modified mNPs reveal a comparable cluster like appearance with sizes between 100 and 200 nm. Scale bars in SEM micrographs indicate a distance of 100 nm. For SPM measurements, samples were prepared by coating freshly cleaved mica with aqueous suspensions of magnetic nanoparticles (0.25 mg/ml in MQ-water). SPM scans were performed using a Multimode V (Veeco, USA). Samples were scanned in non-contact mode with scan rates below 1Hz using standard non-contact mode cantilevers (OMCL-AC160TS, Olympus, Essex, Great Britain). For SEM imaging, samples were prepared by deposition of mNP suspensions (0.25 mg/ml in MQ-water) on cleaned silicon wafers. Wafers were subsequently dried under air stream and gold coated (Auto Fine Coater JSC 1300, Jeol, Akishima, Japan). Nanoparticles on sputtered wafers were imaged with a JSM 7001F Field Emission SEM (Jeol, Akishima, Japan) under high vacuum conditions and room temperature. Accelerating voltage was 20 kV with a focal distance of 10 mm.
(TIF)
