# Correction: The Interplay of Lung Surfactant Proteins and Lipids Assimilates the Macrophage Clearance of Nanoparticles

**DOI:** 10.1371/annotation/3db0e7c6-cf8e-4f1c-a315-9927d201c4be

**Published:** 2012-10-17

**Authors:** Christian A. Ruge, Ulrich F. Schaefer, Jennifer Herrmann, Julian Kirch, Olga Cañadas, Mercedes Echaide, Jesús Pérez-Gil, Cristina Casals, Rolf Müller, Claus-Michael Lehr

There was a formatting error in Equation 1. There is a minus sign missing. The correct equation is:

[Adsorbed amount] = [Control] - [Free amount in the dispersion medium]. 

