# Supplementary figures and images for: The Interplay of Lung Surfactant Proteins and Lipids Assimilates the Macrophage Clearance of Nanoparticles
Source: PLoS One. 2012 Jul 10;7(7):e40775. doi: 10.1371/journal.pone.0040775 (PMC3393659; doi:10.1371/journal.pone.0040775)

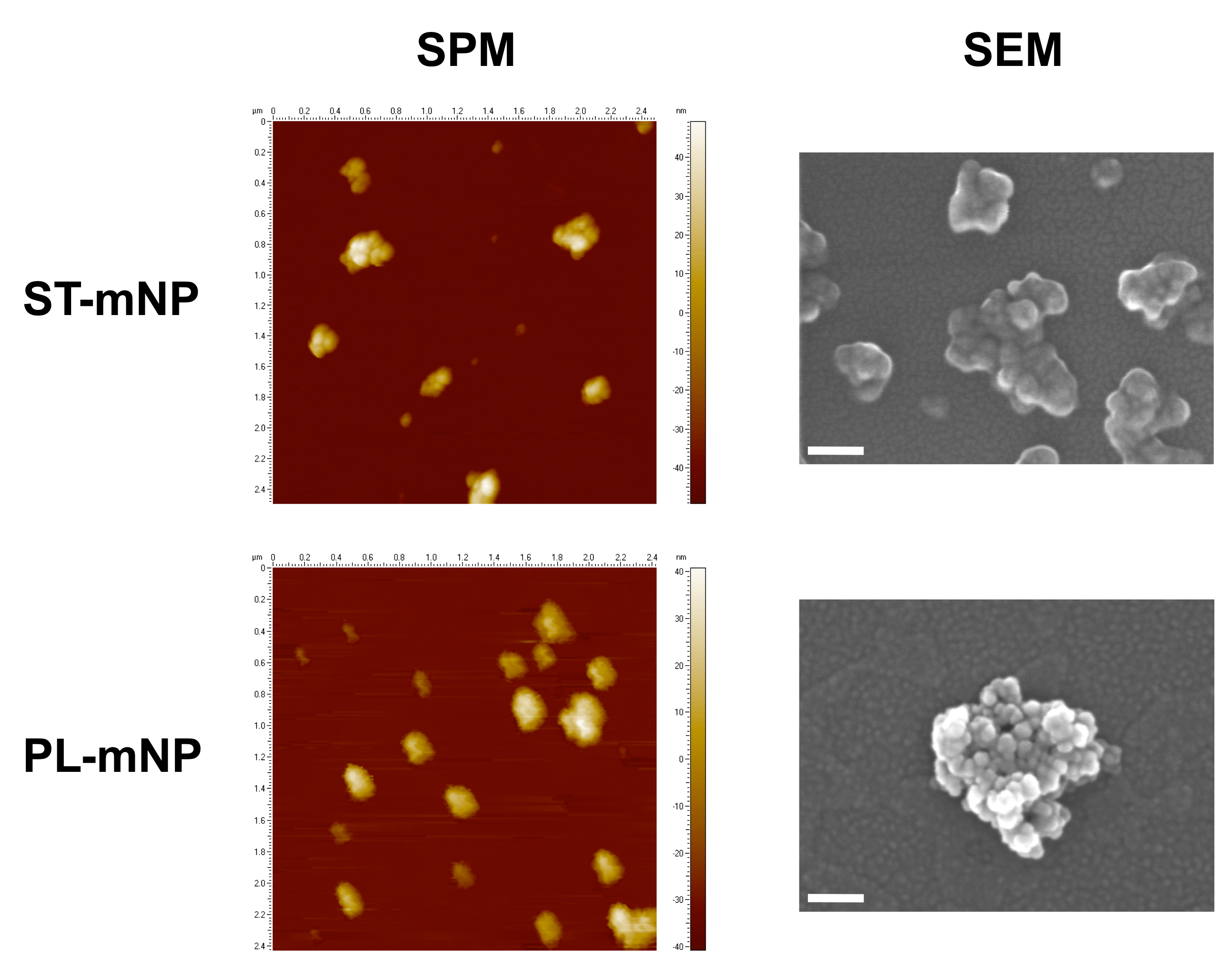

Supplement: Figure S1 — Microscopic images of used magnetic nanoparticles (mNPs). SPM- (left) and SEM-images (right) of starch- (ST) and phosphatidylcholine- modified mNPs reveal a comparable cluster like appearance with sizes between 100 and 200 nm. Scale bars in SEM micrographs indicate a distance of 100 nm. For SPM measurements, samples were prepared by coating freshly cleaved mica with aqueous suspensions of magnetic nanoparticles (0.25 mg/ml in MQ-water). SPM scans were performed using a Multimode V (Veeco, USA). Samples were scanned in non-contact mode with scan rates below 1Hz using standard non-contact mode cantilevers (OMCL-AC160TS, Olympus, Essex, Great Britain). For SEM imaging, samples were prepared by deposition of mNP suspensions (0.25 mg/ml in MQ-water) on cleaned silicon wafers. Wafers were subsequently dried under air stream and gold coated (Auto Fine Coater JSC 1300, Jeol, Akishima, Japan). Nanoparticles on sputtered wafers were imaged with a JSM 7001F Field Emission SEM (Jeol, Akishima, Japan) under high vacuum conditions and room temperature. Accelerating voltage was 20 kV with a focal distance of 10 mm. (TIF) [file pone.0040775.s001.tif]
